# Shared decision making: relevant concepts and facilitating strategies

**DOI:** 10.4178/epih.e2017048

**Published:** 2017-10-30

**Authors:** Jong-Myon Bae

**Affiliations:** Department of Preventive Medicine, Jeju National University Scool of Medicine, Jeju, Korea

**Keywords:** Decision making, Patient participation, Evidence based medicine, Personal autonomy, Health policy

## Abstract

As the paradigm in healthcare nowadays is the evidence-based, patient-centered decision making, the issue of shared decision making (SDM) is highlighted. The aims of this manuscript were to look at the relevant concepts and suggest the facilitating strategies for overcoming barriers of conducting SDM. While the definitions of SDM were discordant, several concepts such as good communication, individual autonomy, patient participants, and patient-centered decision-making were involved. Further, the facilitating strategies of SDM were to educate and train physician, to apply clinical practice guidelines and patient decision aids, to develop valid measurement tools for evaluation of SDM processes, and to investigate the impact of SDM.

## INTRODUCTION

A goal of healthcare currently is evidence-based, patient-centered decision making [1], and accordingly, the importance of “shared decision making (SDM),” in combination with “patient reported outcomes (PRO),” is stressed in the treatment of patients [1-4]. Therefore, healthcare workers should understand the concepts relevant to SDM and be able to apply them while managing patients. This is because good communication between the healthcare workers and patient is also good medical ethics [3]. The objectives of this article are to summarize concepts relevant to SDM and explore the limitations in applying such concepts in clinical practice, as well as ways to overcome them.

## SHARED DECISION MAKING: DEFINITION AND RELEVANT CONCEPTS

Sackett et al. [5] defined evidence-based medicine (EBM) as “the integration of best research evidence with clinical expertise and patient values” in clinical practice. To rephrase it, in EBM decisions are made jointly by physician and patient, based on medical evidence [6].

A theoretical model on medical decision making is categorized into 4 types according to the role of the staff providing the medical service, that is, paternalistic, informed, agent, and shared [7-9]. Of the 4 model types, the shared model is differentiated from the others in that two-way information communication occurs only in it [7,9,10]. The shared model is congruent with the concept of “two-way exchange of information” between physician and patient stressed in the definition of SDM by Charles et al. [11] in their 1997 study. Since the study, diverse definitions of SDM have been proposed and various terms used, causing chaos [4,12,13] (Appendix 1). Furthermore, SDM has been defined in both narrow and wide senses [10].

Over time, various concepts related to the physician-patient relationship (PPR) have been continuously reflected in SDM, which made it difficult for the concept of SDM to be established on a firm ground [12,14]. Important concepts related to PPR can be summarized into the following 4 categories.

First, to establish good PPR requires good communication [4,15]. Especially in healthcare, good communication is critical, because uncertainty cannot be completed excluded in the decision-making process [16]. For good communication to occur, a trusting relationship should be formed between physician and patient using effective conversation techniques [17]. Because physicians can better understand patient problems and clearly identify patient preferences when a trusting relationship is established with the patient [15]. If decisions are made on the basis of the information obtained in such a context, trust is the foundation on which SDM is realized [18,19]. Therefore, for the patient to trust the physician, the latter should summarize the problems at hand in a clear manner, present treatment options for the patient to choose from, and suggest his or her medical opinion [20].

Second, for good communication, patient autonomy should be preserved [21]. Autonomy is a concept related to both a consumercentered culture in society and medical ethics for the protection of patients [10,14]. It has been emphasized to overcome problems arising in the medical culture characterized by paternalistic decision making [10,12,13,22]. When autonomy is guaranteed, patients can exercise the right of self-determination [23] and accept the final outcome of decision making [24]. Considering these points, it is argued that maximizing patients’ capacity for autonomy is an ultimate goal of SDM [10,14].

Third, with patient autonomy being guaranteed, patients should actively participate in the decision-making process [13,22]. To encourage patient participation, the physician should present relevant evidence as he or she summarizes the problems at hand and makes an effort to explain pros and cons of each of the options [25]. When the physician shares relevant information and the patient actively participates in the decision-making process, patients’ treatment satisfaction and compliance increase [26]. Active patient participation should not only be in the decision making process but also in the PRO assessment to evaluate treatment effect [27]. The higher the level of patient participation, the stronger is the patient self-monitoring and the higher the treatment safety [13,24]. Further, it is directly connected to quaternary prevention, the concept of protecting patient safety by preventing overmedicalization [28].

Fourth, for a patient to readily accept the final decision, decision making should be patient-centered, focusing on the characteristics of the patient [6]. In other words, the crux of SDM is that the physician identifies the patient’s personal preference and makes a relevant decision [29]. Indices have been developed to find out patient preference quickly during clinical examination [30]. The idea discussed in this section is linked to value-based medicine, which aims to provide treatment with the maximum cost effectiveness that corresponds to individual patients’ values [2].

## LIMITATIONS IN THE APPLICATION OF SHARED DECISION MAKING AND STRATEGIES TO OVERCOME THEM

Any healthcare workers would welcome SDM if it can be applied in clinical practice, because it brings benefits throughout the field of healthcare [31]. Diverse models have been proposed to apply SDM in clinical practice [3,23,25,32], but there is a large gap between the ideal and reality [3]. Limitations in applying SDM have been pointed out [33] and strategies to overcome them have been suggested [34], from multiple angles. Below, the limitations and overcoming strategies will be discussed from 4 perspectives— physician, patient, healthcare system, and social milieu.

The first perspective is that of physicians, that is, healthcare providers. Visser et al. [35] discussed the limitations in the application of SDM based on the categories of knowledge, attitude, and practice in a systematic review. What is worth noting is that physicians are unlikely to practice SDM in the present context, where they are always out of time; even if they practice SDM, it is predicted that the decisions are likely to be wrong [14,23]. In addition, physicians do not have sufficient understanding of SDM and lack the training to obtain information necessary for decision making [3], and do not understand what patients want [36]. The main strategies to overcome these limitations are education and training of healthcare workers [37]. Towle & Godolphin [38] suggested healthcare workers’ competency needed to practice SDM, and Epstein et al. [32] listed communication skills. These should be included in residency education and training in order to enhance their capacity [22], particularly, to obtain the information necessary in SDM, such as asking questions corresponding to individual patients’ characteristics by organizing essential question items [34] and extracting relevant patient information from various sources including electronic medical records [29]. Furthermore, inter-professional collaboration for SDM would also be required [39].

The second perspective is that of patients or consumers of healthcare service. Longtin et al. [13] pointed out 7 elements that make it difficult for patients to participate in SDM—desire to maintain control, time required to educate and respond to patient, type of illness, personal beliefs, healthcare worker professional specialty, ethnic origin, and insufficient training in patient participation. Moreover, it has been reported that not all patients want to participate in decision making [40], and that cancer patients in particular do not want to participate in treatment decision at an early stage [41]. However, patients are hesitant to participate in SDM due to anxiety they feel because the information provided is insufficient or incorrect [19], and Joseph-Williams et al. [42] have argued that a greater problem is not that patients do not want to participate in the process of SDM but that they cannot. The extent of patient participation depends on patient age, socioeconomic status, disease status, etcetera [43], and accordingly, it is important to strengthen the patient capacity needed for decision making [38]. Therefore, as strategies to overcome the limitations from the patient perspective, clinical practice guidelines [44] and patient decision aids [45] should be developed so that patients can share relevant information within a limited time during clinical examination. In addition, the process of signing the informed consent could be utilized for this purpose [46].

The third perspective is that of the patient care system, where the decision-making process occurs. How well communication with the patient occurs in the SDM process, whether the patient correctly understands the information provided by the physician and conversely, and whether the physician accurately understands the information provided by the patient should be evaluated [26]. Without such an evaluation of the decision making process, both patient and physician could be in doubt about the outcome of decision making [33]. The development and application of valid assessment tools can facilitate the practice of SDM [19]. Current SDM-related assessment tools include the OPTION scale to assess overall SDM process [47]; the HIWQ questionnaire to examine the level of patient participation in SDM [48]; the DESI tool to assess the extent to which the patient accepts the information provided by the physician [20]; the SWD scale to assess satisfaction with the final decision [49]; and COMRADE to assess the outcome from the patient perspective [50]. Validation studies should be conducted on these tools in Korea so that they could be utilized for Korean patients.

The fourth perspective is that of the healthcare delivery system. Only if the health insurance system is improved so that SDM is feasible and the government health authority shows interest can SDM be facilitated [37]. Thus, evidence should be provided to establish laws and policies to facilitate SDM. The recent arguments that SDM enhances the quality of nationwide healthcare systems by decreasing unnecessary medical cost and guaranteeing treatment appropriateness [3,37] are encouraging.

## CONCLUSION

The ultimate goal of SDM is high-quality decision making by patients [10,14]. To achieve the goal, physicians as healthcare providers should play the role of a partner who “shares” relevant information [18]. As SDM has positive effects not only on patients and physicians (i.e., the actors in decision making processes) but also on the government health authority, SDM facilitation must be a primary foundation of national healthcare policies. The establishment and facilitation of SDM in the fee-for-service environment in Korea’s healthcare system is a great challenge to all of physicians, patients, and the government. But at the same time, it will be a critical opportunity to leap forward in improving the quality of the national healthcare.

## Appendix 1. Definitions of shared decision making

**Table:**

Reference	Definition
Charles et al. (1997) [1]	Described as a two-way exchange of information between the parties concerned with the medical decision either from the professional or from a patient‘s point of view
Towle et al. (1999) [2]	Describe decisions that are shared by doctor and patient and informed by best evidence, not only about risks and benefits but also patient specific characteristics and values
Frosch et al. (1999) [3]	Is a process by which patients and providers consider outcome probabilities and patient preferences and reach a health care decision based on mutual agreement
Sheridan et al. (2004) [4]	Is a process in which patients are involved as active partners with the clinician in clarifying acceptable medical options and in choosing a preferred course of clinical care
Briss et al. (2004) [5]	Defined as occurring when a patient and his or her healthcare provider(s), in the clinical setting, both express prefer- ences and participate in making treatment decisions
Joosten et al. (2008) [6]	Defined as an approach in which the clinician and patient go through all phases of the decision-making process together and in which they share the preference for treatment and reach an agreement on treatment choice
Elwyn et al. (2010) [7]	Is a method where clinicians and patients make decisions together using the best available evidence, where patients are encouraged to consider available screening, treatment, or management options and the likely benefits and harms of each
Scholl et al. (2011) [8]	An approach where clinicians and patients communicate together using the best available evidence when faced with the task of making decisions.
