# Supplementary material for: Shared decision making: relevant concepts and facilitating strategies
Source: Epidemiol Health. 2017 Oct 30;39:e2017048. doi: 10.4178/epih.e2017048 (PMC5733387; doi:10.4178/epih.e2017048)
Supplement: Supplementary file 1 [file epih-39-e2017048-supplementary.pdf]

Volume: 39, Article ID: e2017048

https://doi.org/10.4178/epih.e2017048

<특별기고>

공유된 의사결정: 관련 개념과 활성화 전략

Shared Decision Making: Relevant Concepts and Facilitating Strategies

Jong-Myon Bae

Department of Preventive Medicine, Jeju National University Scool of Medicine, Jeju, Korea

Volume: 39, Article ID: e2017048

https://doi.org/10.4178/epih.e2017048

Abstract

As the paradigm in healthcare nowadays is the evidence-based, patient-centered decision making, the issue of shared decision making (SDM) is highlighted. The aims of this manuscript were to look at the relevant concepts and suggest the facilitating strategies for overcoming barriers of conducting SDM. While the definitions of SDM were discordant, several concepts such as good communication, individual autonomy, patient participants, and patient-centered decision-making were involved. Further, the facilitating strategies of SDM were to educate and train physician, to apply clinical practice guidelines and patient decision aids, to develop valid measurement tools for evaluation of SDM processes, and to investigate the impact of SDM.

Key words: Decision making, Patient participation, Evidence based medicine, Personal autonomy, Health policy

Volume: 39, Article ID: e2017048

https://doi.org/10.4178/epih.e2017048

오늘날 보건의료가 지향하는 목표는 근거에 기반한 환자중심의 의사결정 (Evidence-based Patient-centered Decision Making)이다 [1]. 이에 따라 환자 진료에 있어 '환자보고성과' (Patient Reported Outcomes, PRO) 과 함께, '공유된 의사결정' (Shared Decision Making, SDM)의 중요성이 강조되고 있다 [14]. 따라서 보건의료인은 SDM 과 관련한 개념들을 이해하고, 진료과정에서 SDM 개념을 실제로 적용할 수 있어야 한다. 의료인-환자 간의 훌륭한 소통은 의료 윤리 (medical ethics)와도 부합하기 때문이다 [3]. 본 원고는 SDM의 관련 개념들을 정리하고, 진료에 적용하는 과정에서의 한계점들과 이를 극복하기 위한 방안들을 살펴보고자 한다.

1. 공유된 의사결정 (Shared Decision Making): 정의 및 관련 개념들

Sackett et al. [5]은 근거중심의학 (Evidence-based Medicine, EBM)을 임상치료에 있어 '최선의 근거 – 의료진의 전문성 – 환자의 가치란 3가지 요소를 통합하는 것'으로 정의하였다 ("The integration of best research evidence with clinical expertise and patient values'). 이를 다시 말하면 의학적 근거를 바탕으로 의사와 환자 간 상의하여 의사결정을 한다는 것이다 [6].

의사결정에 관한 이론적 모델은 의료서비스를 제공하는 의료진의 역할에 따라 가부장 (paternalistic), 정보제공자 (informed), 중개자 (agent), 공유자 (shared)의 4 종류로 분류한다 [7-9]. 이중 공유자 모델만이 양방향 (two way)으로 정보 교환이 이루어진다는 점에서, 나머지 모델들과 차

Volume: 39, Article ID: e2017048

https://doi.org/10.4178/epih.e2017048

별화 된다 [7,9,10]. 1997년 Charles et al. [11]는 SDM을 정의하면서, 의사와 환자 양자 간의 '양방향 정보 교환' (two-way exchange of information)을 강조한 것과 일맥상통한다. 그러나 이후에 제시된 SDM 정의들은 매우 다양하며, 여러 용어들을 사용하여 혼동스러운 상황이다 [4,12,13] (표 1). 더 나아가 협의와 광의로 구분하여 SDM을 정의하고 있다 [10].

이처럼 SDM의 개념 정립이 이루어지지 않은 이유는 시대를 거치면서 의사-환자 관계 (Physician-patient relationship, PPR)와 관련한 다양한 개념들을 지속적으로 반영해 왔기 때문이다 [12,14]. PPR과 관련한 주요 개념들로 다음 4가지를 정리할 수 있다.

첫째, 원활한 PPR을 이루려면 좋은 소통 (good communication)이 이루어야 한다 [4,15]. 특히 보건의료에 있어 의사 결정 과정에서 불확실성 (uncertainty)을 완전히 배제할 수 없기 때문에 소통은 더욱 중요하다 [16]. 좋은 소통이 되려면 효과적인 대화기법으로 의사-환자간 신뢰(trust) 관계를 형성해야 한다 [17]. 신뢰관계를 확보할 때 환자의 문제를 보다 자세히 이해할 수 있으며, 환자의 선호도 (preference)를 분명히 알 수 있기 때문이다 [15]. 이렇게 알게 된 정보들을 근거로 의사결정을 한다는 점에서, 신뢰 구축은 SDM을 실현할 토대가 된다 [18,19] 따라서 의사는 환자와의 신뢰 구축을 위해 당면 문제를 명확히 정리하고, 선택할 옵션들을 제시한 뒤, 전문적인 제안을 해야 한다 [20].

둘째, 좋은 소통을 만들기 위해서는 환자의 자율성 (autonomy)을 보장해야 한다 [21]. 자율

https://doi.org/10.4178/epih.e2017048

성은 소비자 중심의 사회문화와 환자보호의 의료윤리와 연관된 개념으로 [10,14], 진료현장에서 가부장적 의사결정 문화로 인해 생기는 문제들을 극복하기 위해 강조되었다 [10,12,13,22]. 이런 자율성 확보는 환자에게 자기결정권 (self-determination)을 제공하여서 [23], 의사결정에 따른 최종 결과들을 수용할 수 있게 만든다 [24]. 이러한 점들을 감안할 때, 환자의 자율성 역량을 최대화 시키는 것이 SDM의 궁극적 지향점이라고 주장하고 있다 [10,14].

셋째, 환자의 자율성을 보장한 가운데 의사결정 과정에 적극적으로 환자의 참여 (Patient participants)가 있어야 한다 [13,22]. 환자가 의사결정에 참여하기를 독려하기 위해서 의사는 문제를 정리하면서 관련한 근거들을 제시하고, 여러 선택에 따른 득과 실을 설명하는 노력을 해야 한다 [25]. 이렇게 관련 정보를 공유한 상황에서 의사결정 과정에 환자가 적극 참여할수록 진료에 대한 만족도와 치료 순응도가 올라가기 때문이다 [26]. 환자의 적극적인 참여는, 의사결정 과정뿐만 아니라 치료 후 효과를 평가하기 위한 PRO 측정에도 적극 이루어져야 한다 [27]. 환자의 참여를 높일수록 환자의 자기 모니터링 (self-monitoring)이 강화되면서 치료의 안전성 (safety)이 향상되기 때문이다 [13,24]. 이는 곧 과잉진료를 예방하여 환자의 안전성을 보장하자는 4차 예방 (quaternary prevention) 개념과 연결된다 [28].

넷째, 최종 의사결정한 것을 환자가 적극 수용하게 만드려면 당사자의 개인 특성에 맞춘 환자중심 (patient-centered)의 의사결정이 이루어져야 한다 [6]. 다시 말해서 의사는 환자 개개인의 선호도 (preference)를 알아내어 이에 맞춘 의사결정을 하는 것이 SDM의 핵심 (crux)이다 [29]. 진료현

장에서 짧은 시간 내에 환자의 선호도를 알아내기 위한 지표가 개발되었으며 [30], 개별 환자의 가치에 맞추어 비용 대비 최대 효용의 진료를 하려는 가치중심의학 (value-based medicine)과 연결된다 [2].

2. SDM 적용 한계점들과 이의 극복 전략

SDM이 진료현장에서 적용할 수 있다면 보건의료 전반에 걸쳐 장점을 가진다는 점에서 보건의료인들이라면 모두가 수긍한다 [31]. SDM을 적용하기 위한 다양한 모델들이 제시되었지만 [3,23,25,32], 진료현장의 적용에 이상과 현실의 간극이 크다 [3]. 그동안 SDM 적용상 한계점들이 다각도로 지적되었으며 [33], 이를 극복하는 전략들이 제안되었다 [34]. 이들 한계점과 극복전략을 4가지 측면 – 의사, 환자, 진료시스템, 사회환경 –에서 살펴보고자 한다.

첫째, 의료서비스를 제공하는 의사 측면이다. Visser et al. [35]는 체계적 고찰을 통해 의사 측면에서 지식 (knowledge), 태도 (attitude), 실행 (practice) 범주로 나누어 SDM 적용의 한계점을 제시하였다. 주목할 점으로는 의사들은 시간에 쫓기는 진료 현실상 SDM 수행 가능성이 떨어지며, 적용한다고 해도 잘못된 결정이 될 것이라고 예단하고 있다는 것이다 [14,23]. 또한 SDM 에 대한 이해 부족과 함께 의사결정에 필요한 정보를 확보하기 위한 훈련이 부족하여 [3] 환자가 원하는 것을 제대로 이해하지 못하고 있다 [36]. 이에 대응한 주된 극복 전략은 의료인에 대한 교육 및 훈련이다 [37]. Towle & Godolphin [23]은 SDM 수행에 필요한 의료인의 역량 (competency)을 제시하였고,

Volume: 39, Article ID: e2017048

https://doi.org/10.4178/epih.e2017048

Epstein et al. [32]은 소통을 위한 기법 (communication skills)을 나열하였다. 이를 전공의 교육 및 훈련에 반영하고 역량을 강화해야 할 것이다 [22]. 특히 SDM에 필요한 정보를 얻어낼 수 있도록 필요한 질문 항목들을 정리하여 환자 특성에 맞게 질문할 수 있는 역량을 갖추고 [34], 전자의무기록 등 다양한 정보원에서 관련 정보를 추출해 내는 역량을 갖추도록 한다 [29]. 나아가 SDM을 위한 의사들 간의 협력 (inter-professional collaboration)도 요구된다 [39].

둘째, 의료서비스를 소비하는 환자 측면이다. Longtin et al. [13]은 SDM에서 환자의 참여를 어렵게 하는 7가지 요소들 – 통제 심리, 시간 필요, 질병 특성, 개인 신념, 전문 영역, 윤리 문제, 불충분한 교육 -을 지적하였다. 나아가 모든 환자들이 의사결정에 참여하기를 원하지 않으며 [40], 특히 암 환자일 경우 초기 치료 결정에 참여하기를 원치 않는다는 보고가 있다 [41]. 그러나 제공받는 정보가 부족하거나 잘못되면서 불안감이 생겨 SDM 참여를 주저하는 것이며 [19], SDM의 결정과정에 참여하기를 원하지 않는다가 아니라 참여가 가능하지 않다는 점이 더 문제가 된다고 Joseph-Williams 등이 주장하고 있다 [42]. 참여의 정도는 환자의 연령, 사회경제적 수준, 질병상태 등에 따라 좌우된다는 점에서 [43], 의사결정에 필요한 환자의 역량을 강화 시키는 것이 중요하다 [38]. 따라서 환자측면에서의 한계를 극복하는 전략으로는 임상진료지침 (clinical practice guideline) [44]과 환자조력도구 (patient decision aids) [45]를 개발해서 제한된 진료시간 내에서 환자들이 관련한 정보를 공유할 수 있도록 하며, 환자동의서 (informed consent)를 작성하는 과정을 충분히 활용토록 하는 것이다 [46].

Volume: 39, Article ID: e2017048

https://doi.org/10.4178/epih.e2017048

셋째, 의사결정 과정이 일어나는 진료시스템 측면이다. SDM을 수행하는 과정에서 환자와의 소통이 얼마나 제대로 되고 있는가? 의사가 제공한 정보를 환자가 제대로 이해하고 있는가? 반대로 환자가 제공한 정보를 의사는 얼마나 정확히 파악하고 있는가? 등에 대한 평가 (evaluation)가 필요하다 [26]. 이는 의사결정 과정에 대한 평가가 없다면 환자와 의사 모두에게 의사결정에 대한 의문이 생기기 때문이다 [33]. 따라서 타당한 평가도구의 개발과 이의 적용은 SDM 수행의 촉진자 (facilitator)이다 [19]. SDM과 관련한 평가도구로는 SDM 과정 전반을 점검하기 위한 OPTION 도구 [47], 환자의 SDM 참여 수준을 알아보는 HIWQ 설문서 [48], 의사가 제공한 정보를 환자가 얼마나 수용한가를 알아보는 DESI 도구 [20], 최종 결정에 대한 만족도를 알아보는 SWD 도구 [49], 환자측면에서 성과를 측정하는 COMRADE 도구 [50] 등이 있다. 이들 도구들을 국내 환자에게 적용할 수 있도록 타당성을 확보하는 연구가 필요하다.

넷째, 의료서비스 전달체계 측면이다. SDM을 가능하게 해주는 의료보험체계 개선과 보건당국의 관심이 있어야만 SDM의 적용 활성화가 가능하다 [37]. 다시 말해서, SDM 활성화를 위한 법률 및 정책 수립을 하도록 근거 마련이 필요하다. 최근에 SDM의 활성화는 불필요한 의료 비용을 줄이고, 진료의 적정성을 보장해서 국가 보건의료의 질적 수준을 향상시킨다는 주장 [3,37]은 고무적이다.

3. 결어

SDM의 최종 목표는, 환자가 질 높은 의사결정을 할 수 있도록 하는 것이다 [10,14]. 이를

위하여 의료서비스 제공자인 의사는 관련한 정보들을 '공유'하는 동반자 (partner) 역할을 해야 한다 [18]. SDM을 통해 의사결정의 직접 당사자인 환자와 의사 뿐만 아니라, 보건행정당국까지도 긍정적 영향을 미친다는 점에서 SDM의 활성화는 국가 보건의료 정책의 주요 기조가 되어야 한다. 따라서 행위수가제 (fee-for-services)로 운영되는 국내의 진료 환경에서 SDM의 정착과 활성화는 의사-환자-보건당국 모두에게 주어진 큰 도전이자, 국가보건의료의 질적도약을 이루는 중요한 기회가 될 것이다.

요약문

오늘날 보건의료가 지향하는 목표가 근거에 기반한 환자중심의 의사결정 (Evidence-based Patient-centered Decision Making)인 점에서, 공유된 의사결정 (Shared Decision Making, SDM)의 중요성이 강조되고 있다. 본 원고는 SDM의 관련 개념들을 정리하고, 진료에 적용하는 과정에서의 한계점들과 이를 극복하기 위한 방안들을 살펴보았다.

Key words: 의사결정, 환자참여, 근거중심의학, 개인 자율성, 보건정책

Volume: 39, Article ID: e2017048

https://doi.org/10.4178/epih.e2017048

References

1. Bae JM. Global trends in the use of nationwide big data for solving healthcare problems. J Korean Med Assoc 2014;57:386-390 (Korean).

2. Spatz ES, Elwyn G, Moulton BW, Volk RJ, Frosch DL. Shared decision making as part of value based care: new U.S. policies challenge our readiness. Z Evid Fortbild Qual Gesundhwes 2017;123-124:104-108.

3. Elwyn G, Frosch DL, Kobrin S. Implementing shared decision-making: consider all the consequences. Implement Sci 2016;11: 114.

4. Kasper J, Légaré F, Scheibler F, Geiger F. Turning signals into meaning--'shared decision making' meets communication theory. Health Expect 2012;15:3-11.

5. Sackett DL, Straus SE, Richardson WS, Rosenberg W, Haynes RB. Evidence-based medicine: how to practice and teach EBM. 2nd ed. Edinburgh: Churchill Livingstone; 2000, p. 3.

6. Barry MJ, Edgman-Levitan S. Shared decision making--pinnacle of patient-centered care. N Engl J Med 2012;366:780-781.

7. Charles C, Gafni A, Whelan T. Decision-making in the physician-patient encounter: revisiting the shared treatment decision-making model. Soc Sci Med 1999;49:651-661.

8. Tariman JD, Berry DL, Cochrane B, Doorenbos A, Schepp KG. Physician, patient, and contextual factors affecting treatment decisions in older adults with cancer and models of decision making: a literature review. Oncol Nurs Forum 2012;39:E70-E83.

9. Neuman HB, Charlson ME, Temple LK. Is there a role for decision aids in cancer-related decisions? Crit Rev Oncol Hematol 2007;62:240-250.

10. Cribb A, Entwistle VA. Shared decision making: trade-offs between narrower and broader conceptions. Health Expect 2011; 14:210-219.

11. Charles C, Gafni A, Whelan T. Shared decision-making in the medical encounter: what does it mean? (or it takes at least two to tango). Soc Sci Med 1997;44:681-692.

12. Makoul G, Clayman ML. An integrative model of shared decision making in medical encounters. Patient Educ Couns 2006;60:301-312.

13. Longtin Y, Sax H, Leape LL, Sheridan SE, Donaldson L, Pittet D. Patient participation: current knowledge and applicability to patient safety. Mayo Clin Proc 2010;85:53-62.

14. Martinez KA, Kurian AW, Hawley ST, Jagsi R. How can we best respect patient autonomy in breast cancer treatment decisions? Breast Cancer Manag 2015;4:53-64.

15. Agoritsas T, Heen AF, Brandt L, Alonso-Coello P, Kristiansen A, Akl EA, et al. Decision

aids that really promote shared decision making: the pace quickens. BMJ 2015;350:g7624.

16. Politi MC, Han PK, Col NF. Communicating the uncertainty of harms and benefits of medical interventions. Med Decis Making 2007;27:681-695.
17. Leydon GM, Boulton M, Moynihan C, Jones A, Mossman J, Boudioni M, et al. Cancer patients' information needs and information seeking behaviour: in depth interview study. BMJ 2000;320: 909-913.
18. Chewning B, Bylund CL, Shah B, Arora NK, Gueguen JA, Makoul G. Patient preferences for shared decisions: a systematic review. Patient Educ Couns 2012;86:9-18.
19. Charles C, Gafni A, Whelan T. Self-reported use of shared decision-making among breast cancer specialists and perceived barriers and facilitators to implementing this approach. Health Expect 2004;7:338-348.
20. Frosch DL, Légaré F, Fishbein M, Elwyn G. Adjuncts or adversaries to shared decision-making? Applying the Integrative Model of behavior to the role and design of decision support interventions in healthcare interactions. Implement Sci 2009;4:73.
21. Gulbrandsen P, Clayman ML, Beach MC, Han PK, Boss EF, Ofstad EH, et al. Shared decision-making as an existential journey: aiming for restored autonomous capacity. Patient Educ Couns 2016;99:1505-1510.
22. Butow P, Devine R, Boyer M, Pendlebury S, Jackson M, Tattersall MH. Cancer consultation preparation package: changing patients but not physicians is not enough. J Clin Oncol 2004;22:4401-4409.
23. Elwyn G, Frosch D, Thomson R, Joseph-Williams N, Lloyd A, Kinnersley P, et al. Shared decision making: a model for clinical practice. J Gen Intern Med 2012;27:1361-1367.
24. Davies M, Elwyn G. Advocating mandatory patient 'autonomy' in healthcare: adverse reactions and side effects. Health Care Anal 2008;16:315-328.
25. Elwyn G, Dehlendorf C, Epstein RM, Marrin K, White J, Frosch DL. Shared decision making and motivational interviewing: achieving patient-centered care across the spectrum of health care problems. Ann Fam Med 2014;12:270-275.
26. Kane HL, Halpern MT, Squiers LB, Treiman KA, McCormack LA. Implementing and evaluating shared decision making in oncology practice. CA Cancer J Clin 2014;64:377-388.
27. Shin IS, Chae HJ, Bae JM. Methodology of developing items of patient-reported outcomes using item response theory. Korean J Fam Pract 2016;6:136-139 (Korean).

28. Bae JM, Jamoulle M. Primary care physicians' action plans for responding to results of screening tests based on the concept of quaternary prevention. J Prev Med Public Health 2016;49:343-348.
29. Godolphin W. Shared decision-making. Healthc Q 2009;12:e186-e190.
30. Straus SE, McAlister F. Applying the results of trials and systematic reviews to our individual patients. Evid Based Ment Health 2001;4:6-7.
31. Stacey D, Samant R, Bennett C. Decision making in oncology: a review of patient decision aids to support patient participation. CA Cancer J Clin 2008;58:293-304.
32. Epstein RM, Alper BS, Quill TE. Communicating evidence for participatory decision making. JAMA 2004;291:2359-2366.
33. Elwyn G, Edwards A, Kinnersley P. Shared decision-making in primary care: the neglected second half of the consultation. Br J Gen Pract 1999;49:477-482.
34. Gordon EJ, Butt Z, Jensen SE, Lok-Ming Lehr A, Franklin J, Becker Y, et al. Opportunities for shared decision making in kidney transplantation. Am J Transplant 2013;13:1149-1158.
35. Visser M, Deliens L, Houttekier D. Physician-related barriers to communication and patient- and family-centred decision-making towards the end of life in intensive care: a systematic review. Crit Care 2014;18:604.
36. Cox K, Britten N, Hooper R, White P. Patients' involvement in decisions about medicines: GPs' perceptions of their preferences. Br J Gen Pract 2007;57:777-784.
37. Légaré F, Witteman HO. Shared decision making: examining key elements and barriers to adoption into routine clinical practice. Health Aff (Millwood) 2013;32:276-284.
38. Towle A, Godolphin W. Framework for teaching and learning informed shared decision making. BMJ 1999;319:766-771.
39. Dogba MJ, Menear M, Stacey D, Brière N, Légaré F. The evolution of an interprofessional shared decision-making research program: reflective case study of an emerging paradigm. Int J Integr Care 2016;16:4.
40. Levinson W, Kao A, Kuby A, Thisted RA. Not all patients want to participate in decision making. A national study of public preferences. J Gen Intern Med 2005;20:531-535.
41. Strull WM, Lo B, Charles G. Do patients want to participate in medical decision making? JAMA 1984;252:2990-2994.
42. Joseph-Williams N, Elwyn G, Edwards A. Knowledge is not power for patients: a systematic review and thematic synthesis of patient-reported barriers and facilitators to shared

decision making. Patient Educ Couns 2014;94:291-309.

43. McKinstry B. Do patients wish to be involved in decision making in the consultation? A cross sectional survey with video vignettes. BMJ 2000;321:867-871.

44. van der Weijden T, Pieterse AH, Koelewijn-van Loon MS, Knaapen L, Légaré F, Boivin A, et al. How can clinical practice guidelines be adapted to facilitate shared decision making? A qualitative key-informant study. BMJ Qual Saf 2013;22:855-863.

45. Bae JM. Development and application of patient decision aids. Epidemiol Health 2015;37:e2015018.

46. Spatz ES, Krumholz HM, Moulton BW. The new era of informed consent: getting to a reasonable-patient standard through shared decision making. JAMA 2016;315:2063-2064.

47. Elwyn G, Edwards A, Wensing M, Hood K, Atwell C, Grol R. Shared decision making: developing the OPTION scale for measuring patient involvement. Qual Saf Health Care 2003;12:93-99.

48. Xie B, Wang M, Feldman R, Zhou L. Exploring older and younger adults' preferences for health information and participation in decision making using the Health Information Wants Questionnaire (HIWQ). Health Expect 2014;17:795-808.

49. Holmes-Rovner M, Kroll J, Schmitt N, Rovner DR, Breer ML, Rothert ML, et al. Patient satisfaction with health care decisions: the satisfaction with decision scale. Med Decis Making 1996;16: 58-64.

50. Edwards A, Elwyn G, Hood K, Atwell C, Robling M, Houston H, et al. Patient-based outcome results from a cluster randomized trial of shared decision making skill development and use of risk communication aids in general practice. Fam Pract 2004;21:347-354.

Volume: 39, Article ID: e2017048

https://doi.org/10.4178/epih.e2017048

Table 1. Definitions of shared decision making

Reference*	Definition
Charles (1997) [1]	described as a two-way exchange of information between the parties concerned with the medical decision either from the professional or from a patient's point of view.
Towle (1999) [2]	describe decisions that are shared by doctor and patient and informed by best evidence, not only about risks and benefits but also patient specific characteristics and values.
Frosch (1999) [3]	is a process by which patients and providers consider outcome probabilities and patient preferences and reach a health care decision based on mutual agreement.
Sheridan (2004) [4]	is a process in which patients are involved as active partners with the clinician in clarifying acceptable medical options and in choosing a preferred course of clinical care.
Briss (2004) [5]	defined as occurring when a patient and his or her healthcare provider(s), in the clinical setting, both express preferences and participate in making treatment decisions.
Joosten (2008) [6]	defined as an approach in which the clinician and patient go through all phases of the decision-making process together and in which they share the preference for treatment and reach an agreement on treatment choice.
Elwyn (2010) [7]	is a method where clinicians and patients make decisions together using the best available evidence, where patients are encouraged to consider available screening, treatment, or management options and the likely benefits and harms of each.
Scholl (2011) [8]	an approach where clinicians and patients communicate together using the best available evidence when faced with the task of making decisions.

References

1. Charles C, Gafni A, Whelan T. Shared decision-making in the medical encounter: what does it mean? (or it takes at least two to tango). Soc Sci Med 1997;44:681-692.

Volume: 39, Article ID: e2017048

https://doi.org/10.4178/epih.e2017048

2. Towle A, Godolphin W. Framework for teaching and learning informed shared decision making. BMJ 1999;319:766-771.
3. Frosch DL, Kaplan RM. Shared decision making in clinical medicine: past research and future directions. Am J Prev Med 1999;17:285-294.
4. Sheridan SL, Harris RP, Woolf SH; Shared Decision-Making Workgroup of the U.S. Preventive Services Task Force. Shared decision making about screening and chemoprevention. a suggested approach from the U.S. Preventive Services Task Force. Am J Prev Med 2004;26:56-66.
5. Briss P, Rimer B, Reilley B, Coates RC, Lee NC, Mullen P, et al. Promoting informed decisions about cancer screening in communities and healthcare systems. Am J Prev Med 2004;26:67-80.
6. Joosten EA, DeFuentes-Merillas L, de Weert GH, Sensky T, van der Staak CP, de Jong CA. Systematic review of the effects of shared decision-making on patient satisfaction, treatment adherence and health status. Psychother Psychosom 2008;77:219-226.
7. Elwyn G, Laitner S, Coulter A, Walker E, Watson P, Thomson R. Implementing shared decision making in the NHS. BMJ 2010;341:c5146.
8. Scholl I, Koelewijn-van Loon M, Sepucha K, Elwyn G, Légaré F, Härter N, et al. Measurement of shared decision making: a review of instruments. Z Evid Fortbild Qual Gesundhwes 2011;105:313-324.
